# From Crisis to Clarity: Retropupillary Iris-Claw Lens and Iris Cerclage Techniques for Post-traumatic Cataract Rehabilitation

**DOI:** 10.7759/cureus.75416

**Published:** 2024-12-09

**Authors:** Nisha Ahuja, Lakshita Maherda, Karishma Dubey

**Affiliations:** 1 Ophthalmology, Sankara Academy of Vision, Anand, IND; 2 Ophthalmology, Sankara Eye Hospital, Anand, IND

**Keywords:** cataract, cerclage, pupillary, subluxated lens, traumatic mydriasis

## Abstract

This study details two cases of traumatic cataracts with a history of blunt trauma. Both presented with progressive vision loss, mydriasis, and zonular dialysis. The surgical intervention involved complete cataractous lens removal, anterior vitrectomy, iris cerclage with 10-0 prolene sutures, and retropupillary iris-claw lens fixation. Postoperative outcomes showed significant visual acuity improvements: vision reached 6/12 with correction in both cases. These findings suggest that combining iris cerclage with retropupillary iris-claw lens implantation is a safe, faster, and effective strategy for managing traumatic cataracts, offering substantial visual rehabilitation with minimal complications.

## Introduction

Blunt eye trauma can lead to injury of the iris sphincter muscle, either focal or diffuse, resulting in varying degrees of traumatic mydriasis, and anisocoria with or without lens subluxation [1]. Traumatic mydriasis along with a cataract can cause symptoms like glare, photophobia, and reduced visual acuity. Several surgical techniques have been described for the repair of a wide, nonreactive post-traumatic pupil, including the use of single or multiple interrupted iris sutures, running sutures with forceps for iris manipulation, and the single-pass four-throw method, among others [2-4]. The aim of these procedures is to restore the pupil’s appearance and function as closely as possible to its natural state. Another technique, iris cerclage, is employed to treat persistent mydriasis caused by diffuse iris sphincter dysfunction and typically results in a more round pupil, providing better control over pupil size. In this case report, in both cases, the patients underwent a combined iris cerclage pupilloplasty along with retropupillary insertion of an iris-claw lens following the removal of cataractous subluxated lens in toto. This resulted in good postoperative outcomes, including anatomical position, best-corrected visual acuity (BCVA), minimal glare, and photophobia.

## Case presentation

Case 1

A 55-year-old male presented with gradual, progressive, painless diminish of vision in his right eye over the past year, following blunt trauma with a wooden stick. On examination, visual acuity in the right eye was PL (perception of light) present and PR (projection of rays} accurate in all four quadrants, while the left had an uncorrected vision of 6/60, N18.

**Figure 1:**
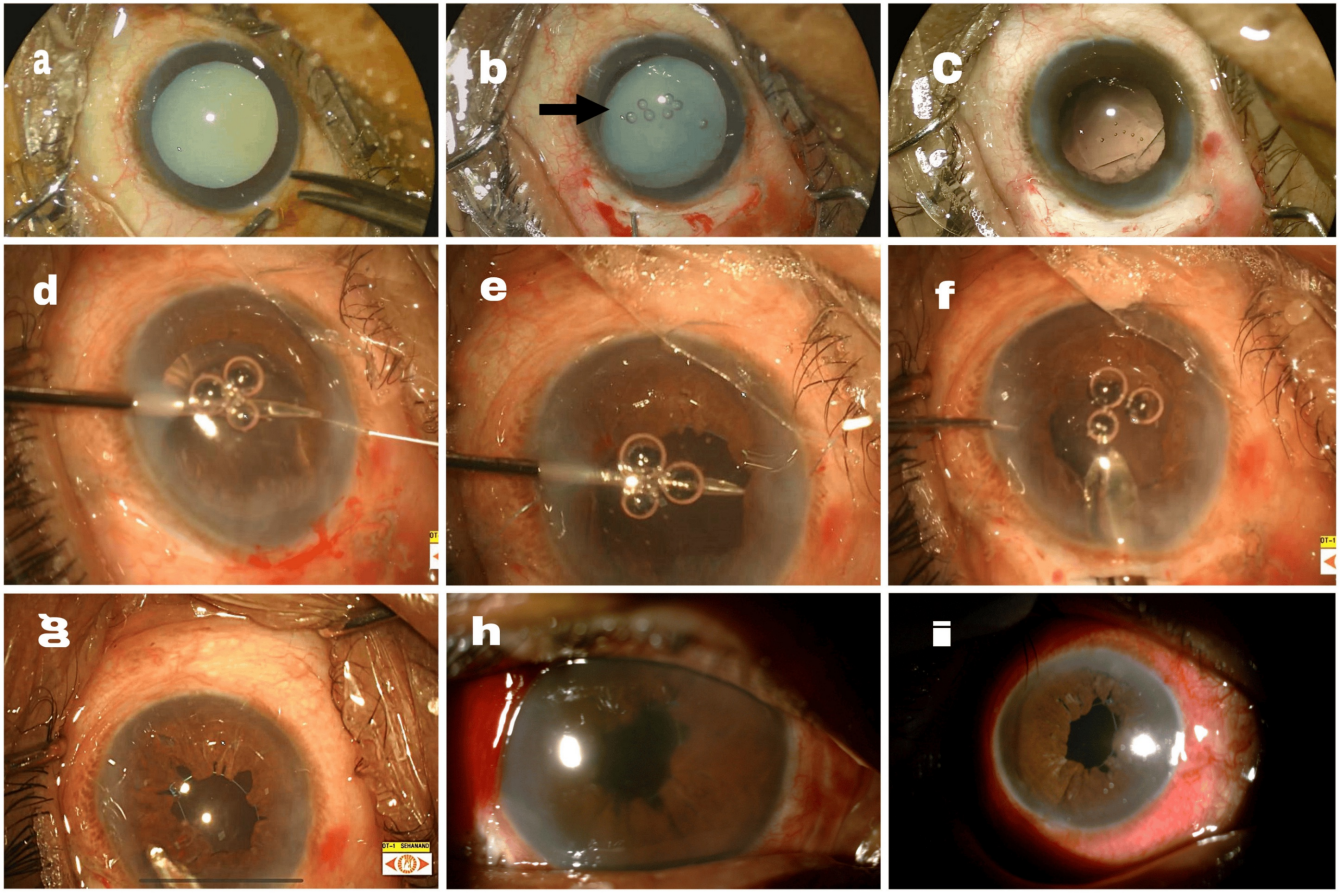
Subluxated hypermature cataract with traumatic mydriasis operated by pupillary cerclage and retropupillary iris-claw fixation a: A hypermature cataract is shown with accompanying traumatic mydriasis. b: After injecting viscoelastic in the anterior chamber through the side port, subluxation in the superonasal quadrant (black arrow) was noted. c: The image shows aphakia following the complete removal of the cataractous lens and automated anterior vitrectomy. d: A double-headed straight prolene suture (10-0) has been passed from the temporal side covering the inferior 180 degrees. e: A suture has been threaded through the upper 180 degrees of the iris, with the knot secured superonasally. f: A retropupillary iris-claw lens has been successfully implanted. g: The viscoelastic material has been completely removed, intracameral moxifloxacin was administered, and the anterior chamber was formed. h: On the first postoperative day, corneal edema, along with cells and flare in the anterior chamber, were noted. i: By the 15th postoperative day, the cornea was clear with a quite anterior chamber.

The anterior segment of the right eye showed a subluxated hypermature cataract with traumatic mydriasis, and the left eye had nuclear sclerosis grade 1 (Figure 1). Other anterior segment findings for both eyes were normal. B-scan imaging was normal for the right eye, and the fundus was normal for the left eye.

The surgical intervention included cataractous lens removal in-toto followed by anterior vitrectomy, iris cerclage with 10-0 prolene sutures (double-headed straight), and retropupillary iris-claw fixation. Postoperatively, on the first day, the vision in the right eye was 2/60 with an improvement of 3/60 with a pinhole. The anterior segment showed corneal edema, 4+ cells, 3+ flare, and membrane, with a hazy fundus view. 

With appropriate topical and systemic medications, the patient’s condition improved significantly by the 15th postoperative day, with visual acuity improving to 6/12, N6 with correction, and a clear fundoscopic view. 

Case 2

A 55-year-old male presented with gradually progressive painless diminish of vision in both eyes for one year. There was a history of trauma one and a half years ago. 

**Figure 2:**
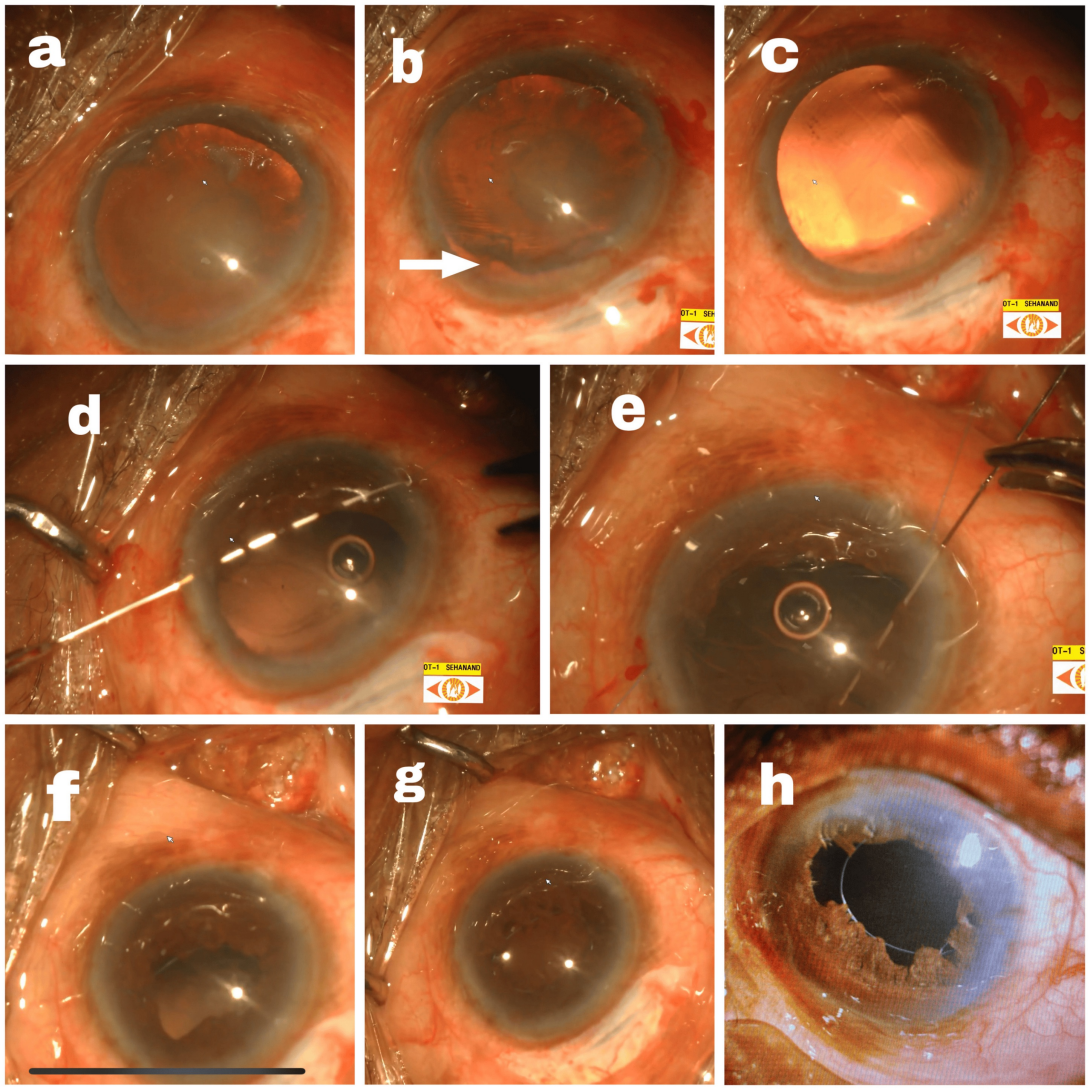
Subluxated immature cataract with traumatic mydriasis operated by pupillary cerclage and retropupillary iris-claw fixation a: A brown cataract is shown with accompanying traumatic mydriasis. b: After accessing the anterior chamber through a side port, the image reveals a subluxation in the superotemporal quadrant (white arrow), alongside traumatic mydriasis. c: The image shows aphakia following the complete removal of the cataractous lens after anterior vitrectomy. d: A double-headed straight prolene suture (10-0) has been passed from the temporal side covering the inferior 180 degrees. e: A suture has been threaded through the upper 180 degrees of the iris. f: Knot was tied externally and secured temporally. g: A retropupillary iris-claw lens has been successfully implanted. The viscoelastic material has been completely removed, intracameral moxifloxacin was administered, and the anterior chamber was restored. H: Postoperative image with clear cornea, quite anterior chamber, and iris-claw lens in situ.

His visual acuity in the right eye was 6/60, N12, and in the left eye was 2/60, N36. Anterior segment examination of the right eye showed a brown cataract (nuclear sclerosis grade 4 with posterior subcapular cataract) and the left eye had an inferiorly subluxated brown cataract (nuclear sclerosis grade 4 with posterior subcapsular) with traumatic mydriasis (Figure 2). Fundoscopy for the right eye was normal with a hazy view. B-scan was normal for the left eye.

The surgical intervention included cataractous lens removal in toto followed by anterior vitrectomy, iris cerclage with 10-0 prolene sutures (double-headed straight), and retropupillary iris-claw fixation. On the first postoperative day, vision was 6/36 improving to 6/24 with a pinhole with 1+ cells in the anterior chamber. All other findings were normal. On day 15, his BCVA (best-corrected visual acuity) was 6/12 with -1.75 sphere and -1.5 at 1800, N8 with +2.75 D sphere.

## Discussion

Blunt trauma to the eye can have varied presentation including cataract formation with or without subluxation or dislocation. Injury to the iris can lead to mild to severe iridodialysis, traumatic mydriasis, and anisocoria [5]. Various methods have been outlined for surgically repairing a wide post-traumatic mydriatic pupil. These methods include single or multiple interrupted iris sutures, running sutures using forceps for iris manipulation, the single-pass four-throw technique, and other approaches [6]. Iris cerclage includes multiple small bites taken with 10-0 prolene sutures on the pupillary margin for 3600 reducing pupillary size [7]. Many methods have been used for IOL implantation in eyes lacking capsular support to reduce post-operative high anisometropia like ACIOL [8], SFIOL [9], and Iris claw (retropupillary and prepupillary) lenses [10]. Although scleral fixated posterior chamber IOL offers the lens in the anatomical position, at the same time, it is more time-consuming and technically difficult [7].

Retropupillary iris-claw lenses have the advantages of quick procedure, no requirement for sutures, reversibility, and less learning curve without significant endothelial cell loss and offer the lens very close to its anatomical position [7]. These patients presented with HMSC and brown cataracts, respectively, and zonular dialysis, resulting in lens subluxation. We addressed this by performing a complete lens removal. To manage the traumatic mydriasis, we carried out a pupilloplasty using a 10-0 prolene suture for the iris cerclage to reconfigure the pupil. This allowed us to successfully implant a retropupillary iris-claw lens with a good visual outcome. Thus, retropupillary iris-claw implantation along with iris cerclage pupilloplasty in eyes lacking capsular or zonular support with traumatic mydriasis is a safe, faster, and effective procedure with good visual rehabilitation and fewer complications.

## Conclusions

This case series highlights the effectiveness and safety of combining iris cerclage pupilloplasty with retropupillary iris-claw lens implantation for managing traumatic cataracts and persistent mydriasis in eyes with zonular dialysis or aphakia. Both patients experienced remarkable visual recovery, with excellent anatomical positioning of the iris-claw lens and minimal postoperative complications. This innovative approach offers a fast, minimally invasive solution for complex traumatic cataract cases, significantly improving visual function while addressing common symptoms such as glare and photophobia. The combination of iris cerclage and retropupillary lens implantation provides a promising, efficient alternative to traditional techniques, ensuring optimal visual rehabilitation and superior patient outcomes in eyes lacking capsular support.
